# Electromagnetic Interference (EMI) Shielding Performance and Photoelectric Characteristics of ZnS Infrared Window

**DOI:** 10.3390/ma18051067

**Published:** 2025-02-27

**Authors:** Liqing Yang, Rongxing Guo, Fei Gao, Yongmao Guan, Mengwen Zhang, Pengfei Wang

**Affiliations:** 1 Henan Key Laboratory of Aeronautical Materials and Application Technology, Zhengzhou University of Aeronautics, Zhengzhou 450046, China; guorongxing@zua.edu.cn; 2 National Key Laboratory of Ultrafast Optical Science and Technology, Xi’an Institute of Optics and Precision Mechanics, Chinese Academy of Sciences (CAS), Xi’an 710119, China; yangliqing@opt.ac.cn (L.Y.); gaofei1980@opt.ac.cn (F.G.); guanyongmao@opt.ac.cn (Y.G.)

**Keywords:** infrared optical window, metal mesh, crack template, electromagnetic shielding

## Abstract

ZnS material shows great application prospects in fields such as infrared windows, fairings, and lenses. In this study, a crack template method was developed to prepare gold meshes with random structures on ZnS optical window. The crack template and gold meshes structures were designed from a completely new perspective focusing on the period and line width ratio. Then, four different structural parameters of the gold mesh were fabricated using the crack template method, their ratios of the aperture to line width were 16.1, 17.4, 18.0, and 19.0. The templates’ morphology and structural traits were examined via optical and laser confocal microscopy. The sample with a ratio of aperture to line width of 16.0 had the best connectivity and the highest coverage, at 15.33%, while the sample with a ratio of aperture to line width of 19.0 had the lowest coverage, at 11.64%. Gold meshes were deposited using these templates, where an increase in the aperture-to-line width ratio resulted in average transmittances of 57.1% and 63.2% over the 2–10 μm range. The electromagnetic shielding efficiency surpassed 22.5 dB within the 1–18 GHz range, while the 1#-mesh, with an aperture-to-line width ratio of 16.0, achieved 33.2 dB at 1 GHz. This research endeavor contributes significantly to advancing the understanding of the ZnS glass’ optoelectric performance and enhances their potential for practical applications.

## 1. Introduction

Infrared windows play an important role in infrared propagation, protecting internal detection signals, imaging systems, and dissipating heat generated by internal components during service. They are widely used in many fields such as space communication, remote sensing detection, and optoelectronic countermeasures [1,2,3,4,5]. With the constant development of infrared technology, the working band of various infrared systems has gradually expanded from near-infrared and mid-infrared to far-infrared bands. The 3–5 μm and 8–14 μm bands are known as the “atmospheric window zone” due to their good transmittance to the atmosphere and minimal attenuation of radiation intensity. Therefore, the optical materials serving in the range of 3–5 μm mid-infrared and 8–14 μm far-infrared wavelengths have become important optical window materials. Commonly used infrared window materials include crystal materials such as Ge, zinc sulfide, zinc selenide, sapphire, as well as glass materials such as barium gallo-germanate (BGG) glass, gallium oxyfluoride (FGa) glass, and Zr-Ba-La-Al-Na (ZBLAN) glass. Among many infrared materials, ZnS material has an ultra-wide infrared transmission range, which can be transparent in an extremely wide energy range, with a very large transmission from visible wavelengths to just over 12 μm [6,7]. It monopolizes the mid to far infrared region that other optical glasses cannot pass through. Sulfur-based glass has a series of excellent optical properties such as extremely high nonlinear refractive index, adjustable composition, and low phonon energy [6]. It can be directly processed into infrared optical lenses through precision molding, its preparation and processing costs are much lower than single crystal germanium, and its size is not limited. So, it is considered as the core material of the new generation of infrared optical windows [8].

Then, in the aerospace environment with information technology rapidly developing, various infrared imaging systems also face serious electromagnetic interference (EMI), but these optical windows cannot be directly coated with opaque electromagnetic shielding materials, and it has become the weakest part in electromagnetic protection. Therefore, there is an urgent need for optical windows with high transparency, anti-electromagnetic shielding reinforcement.

Numerous types of transparent EMI shielding films exhibiting excellent shielding performance have been reported [9,10,11,12,13,14,15,16,17,18]. Among the novel EMI shielding materials, a continuous transparent conductive film, indium tin oxide (ITO), has played a significant role in transmitting visible light and shielding long wave EMI. However, due to the proximity of the passband, low transmittance of the substrate material, and limitations of continuous film materials, in terms of transmitting infrared light and shielding long wave EMI [19,20,21], the metal mesh is still the only choice at present.

Excellent conductivity and good electromagnetic shielding capability are provided by metal mesh. Metal mesh films have special benefits in electrical conductivity, especially those composed of copper, silver, and gold [22,23,24,25]. Specifically, the random transparent metal mesh provides high optical transmittance and effective electromagnetic shielding, while also mitigating or even eliminating the starlight effect in the optical window [26,27,28,29,30]. Clearer and more precise observed images are the outcome of this development, which also improves the optical system’s imaging capability. It follows that there have been numerous research results on metal mesh shielding films for optical windows in the visible light band, but there is relatively little research on optical windows in the infrared band. To date, metal mesh films have predominantly been fabricated using micro-nanofabrication technique which are costly and involve complex processes, such as laser direct writing [31], ion beam etching [32], ultraviolet lithography [12], electric field-driven microscale 3D printing [33], and nanoimprint lithography [34]. In this study, we developed an IR transparent gold (Au) mesh optical window on a ZnS substrate. The irregular structure metal meshes were fabricated by the crack template methods based on a completely new perspective focusing on the period and line width ratio, which provided efficient and stable EMI shielding properties over a broad frequency spectrum and maintained high optical transparency in the infrared range. These findings serve as a theoretical foundation and technical reference for the engineering implementation of infrared transparent electromagnetic shielding optical windows.

## 2. Structure Design and Simulation

**Figure 1:**
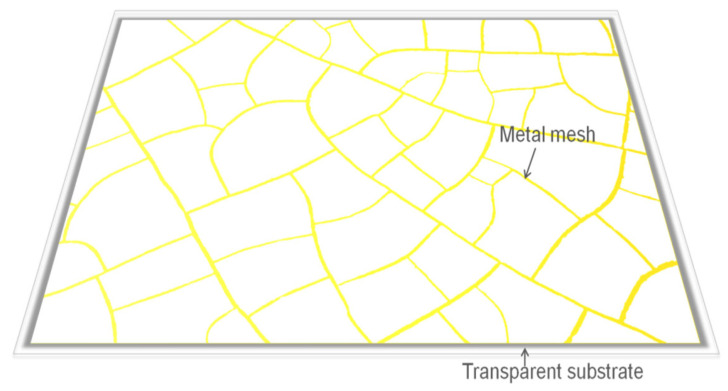
Random irregular structure of the IR transparent EMI shielding window.

Figure 1 shows the structure of the IR transparent EMI shielding window. Considering the balance between optical transmission and electromagnetic shielding performance. In theory, metal mesh transmittance is determined by its coverage ratio, incorporating both the average wire width and mesh spacing. The optical transmission (T) of different metallic meshes can be calculated using Equation (1) where a represents the average line width and g represents the average periodic length [35]. This extended model also considers multi-layer mesh structures, with the number of layers represented by n. where Ts is the transmittance of the substrate.

(1) $$T=T_{s}{\left(1-\frac{W}{W+g}\right)}^{2n}$$

**Figure 2:**
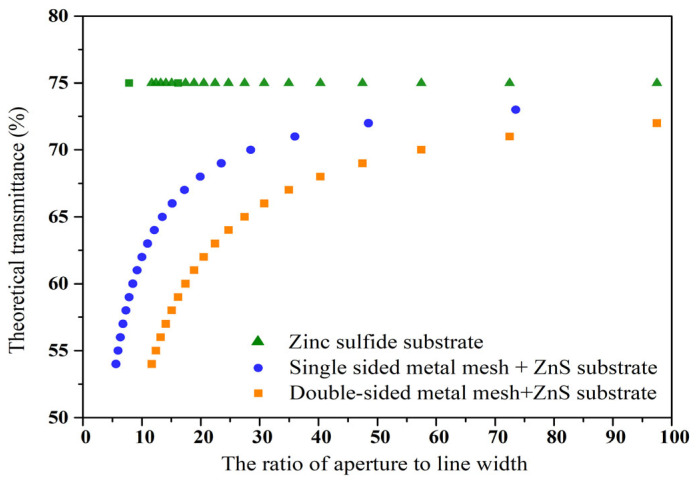
Theoretical transmittance at 10 μm of the ZnS windows with the different Au meshes.

The transmittance of ZnS glass itself is approximately 75% (in the wavelength 2–10 μm) [36]. The transmittance variation curves of the substrate with single-sided metal mesh and double-sided metal mesh are calculated using Equation (1) for different ratios of aperture to line width, as shown in Figure 2.

In metal mesh films, EMI shielding primarily results from electromagnetic wave reflection. Therefore, the shielding effectiveness depends on the metal mesh film’s electrical conductivity and can be further described as follows [37]:

(2) $$SE=20lg\left(1+\frac{Z_{0}}{2R_{s}}\right)$$

where $Z_{0}=377\;\Omega$ is the wave impedance of free space and $R_{s}$ is the sheet resistance. Low sheet resistance materials generally provide enhanced EMI shielding, rendering low-resistance metal mesh films highly efficient for shielding applications.

Sheet resistance is determined by conductivity σ and skin depth δ, remaining independent of thickness (t), width (w), and mesh spacing (g). It can be evaluated by an Equation (3) [38].

(3) $$R_{s}=\frac{1}{\sigma \delta (1-e^{-t/\delta })}\frac{g}{2w}$$

where σ is the conductivity (S/m), δ is the skin depth (μm), and t is the thickness of metal mesh film (μm).

Skin depth (δ) is related with the frequency (f) of incident plane wave and conductivity (σ) of metal mesh film, and it can be calculated by a function Equation (4).

(4) $$\delta =\sqrt{\frac{1}{\pi f\mu \sigma }}$$

where µ is the permeability (H/m), µ = 4π × 10^−7^ H·m^−1^, σ = 4.17 × 10^7^ S/m, and the EMI frequency band is from 1 to 18 GHz. According to the simulation calculation of Equations (2)–(4), the electromagnetic shielding effectiveness of the metal mesh with a certain aperture and line width (*g*/*w*) can be theoretically estimated.

## 3. Materials and Methods

### 3.1. Metal Mesh Preparation

**Figure 3:**
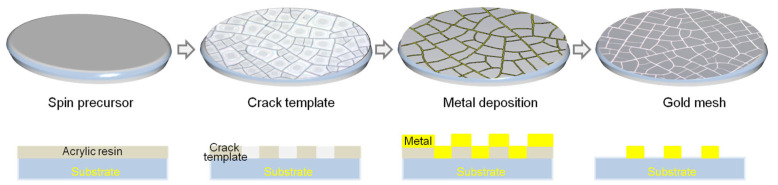
Schematic diagram of the preparation process of gold mesh.

After optical polishing, zinc sulfide with a thickness of 5 mm was chosen as the clear substrate for creating the Au meshes coated with EMI shielding infrared windows. Acetone, isopropanol, and deionized water were used to ultrasonically clean the substrates for two minutes each. The fractured templates were derived from a colloidal dispersion of acrylic resin. The acrylic resin colloidal dispersion was synthesized in-house (see Supplementary Information). Drops of the colloidal dispersion based on acrylic resin were spin coated onto the substrate for 60 s and then dried at room temperature (25 °C) with 50% humidity to form crack template. A 300 nm-thick conductive Au film was deposited onto the cracked template via an e-beam evaporator (ZZS110, Chengdu Xingnanyi Vacuum Equipment Co., Ltd, Chengdu, China) at room temperature under a base vacuum of 5.4 × 10^−4^ Pa, anode voltage of 200 V, and anode current of 1.5 A. The cracked template was removed via lift-off by immersing and sonicating the sample in 2-Acetoxy-1-methoxypropane (PGMEA) for 3–5 min [39]. Finally, a conductive Au mesh were produced. The gold mesh preparation process is depicted in Figure 3.

### 3.2. Microscopic Study

With the aid of an optical microscope (BX53M, Olympus Corporation, Tokyo, Japan), the morphologies of the Au meshes and fractured templates were investigated. After a 12-h drying phase, the film thicknesses were assessed using a probe profilometer (Dektak XT, Bruker, Bill Rika, MA, USA). The surface geometry of the cracked templates was measured using an Olympus OLS4100 laser confocal microscope. Custom image processing software was used to process these micrographs in order to analyze the width and size of each fracture zone.

### 3.3. Optoelectric Study

A FT&IR spectrometer (VERTEX70, Bruker, Bill Rika, MA, USA) was used to measure the spectrum dependencies of the optical transmittance of the Au meshes in the 2–15 μm. A four-probe head was used to measure the sheet resistance (ST2258C, Suzhou Jingge Electronic Co., Ltd., Suzhou, China).

### 3.4. EMI Shielding Measurements

Waveguide-to-coaxial adapters and a Vector Network Analyzer (E5080B, Keysight Technologies, Santa Clara, CA, USA) were used to assess EMI shielding effectiveness (SE) in the frequency range of 1–18 GHz in order to determine the scattering parameter (S_21_).

## 4. Results

### 4.1. Morphology and Structure of Metal Mesh

**Figure 4:**
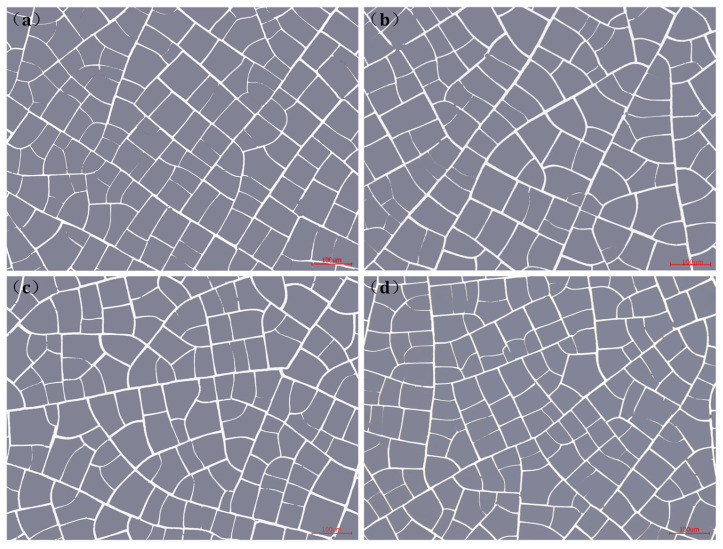
The morphology images of the Au mesh (**a**–**d**) *g*/*w* is 16.0, 17.4, 18.1, and 19.0.

**Figure 5:**
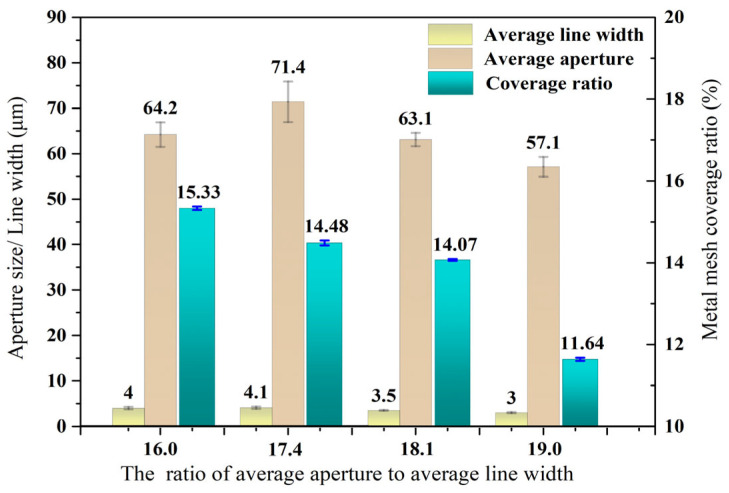
The values of aperture and line width of the Au mesh.

Figure 4 presents the Au mesh morphology observed via optical microscopy, where most meshes exhibit a quadrilateral shape. The metal lines are uniform and have good connectivity with each other. We selected four samples (1#–4#) with a ratio of *g*/*w* from 16.0 to 19.0 and used a self-developed image analysis software to statistically analyze the *g*/*w* values of the metal mesh. For each sample, 15 microscopic images were captured at different positions to determine the average aperture and line width values, as illustrated in Figure 5. At the same time, the coverage of each sample Au mesh on the substrate was calculated through the software. Among them, the sample with *g*/*w* of 16.0 had the highest coverage, at 15.33%, while the sample with *g*/*w* of 19.0 had the lowest coverage, at 11.64%.

**Figure 6:**
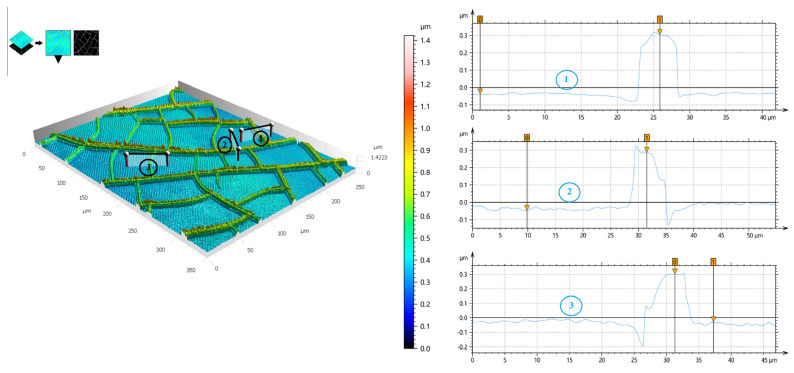
The thickness profile of the Au mesh. ①②③ is three different test sites.

Figure 6 presents the thickness results of the Au mesh. The same sample (1#) was tested at three different positions, and the maximum thickness at all three test points was approximately 0.3 μm, demonstrating the high uniformity of the vapor deposition process.

### 4.2. Optical and Electrical Properties of ZnS EMI Shielding Windows

**Figure 7:**
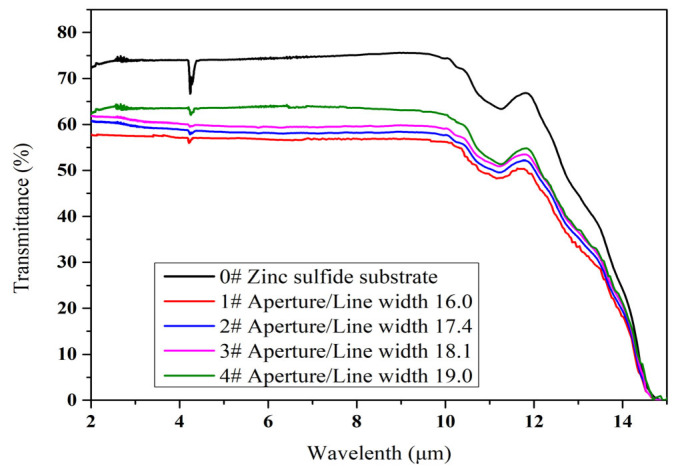
The optical transmittance in the ranges of 2–15 μm of the ZnS windows.

Figure 7 shows the variation in the transmittance spectra of the four samples from 2 μm to 15 μm wavelengths. Among those metal mesh samples (ZnS glass coated with Au mesh), the sample corresponding to the minimum ratio of *g*/*w* shows the highest transmittance over the entire spectrum. As the ratio of *g*/*w* increases, the average transmittance from 2 μm to 10 μm is increased continuously from 57.1% to 63.2%, as shown in Figure 7. This is consistent with the theoretical prediction results in Section 2. As the ratio of *g*/*w* increases, the corresponding transmittance will also increase. This is because, as the aperture size of the Au mesh increases and the line width narrows, the amount of light transmitted will naturally increase.

**Figure 8:**
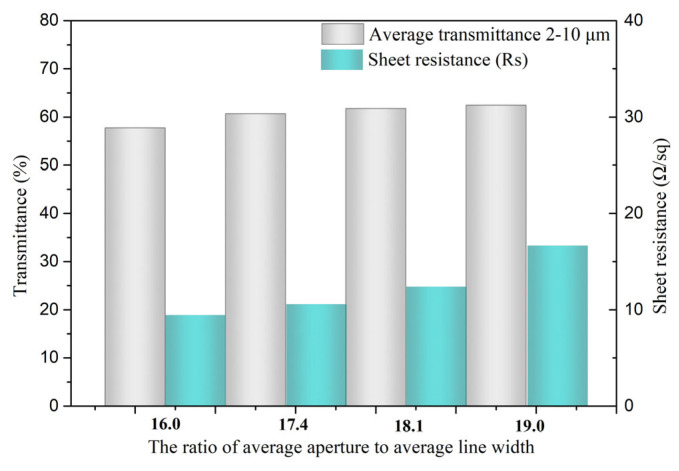
Sheet resistances and transmittance with the ratio of aperture and line width.

The primary goal of creating metal mesh on substrates for electromagnetic shielding optical windows is to minimize surface resistance while maximizing light transmission efficiency. Figure 8 illustrates how the ratio of aperture to line width affects the sheet resistances. The sheet resistance is at its lowest, 9.5 Ω/sq, when the ratio is 16.0. Equation (3) in Section 2 states that the sheet resistance rises as the aperture to line width ratio increases. It also depends on the lattice characteristics, deposition circumstances, and flaws in the lines and junctions. Therefore, rather than both having optimal values, an optimal balance can only be reached between transmittance and surface resistance. Another formula, $R_{□}=\xi \frac{\sigma }{t\cdot r_{c}}$, from earlier literature reports also yields the same calculation result [40]. In this equation, ξ represents a correction factor influenced by lattice properties, deposition conditions, and imperfections at lines and junction, while σ denotes the metal’s electrical resistivity, t is its thickness, and r_c_ is its coverage ratio. As the coverage ratio rises from 11.64% to 15.33% in this work, the Au meshes’ sheet resistance decreases from 16.7 Ω/sq to 9.5 Ω/sq.

### 4.3. EMI Shielding Properties of Au Meshes

**Figure 9:**
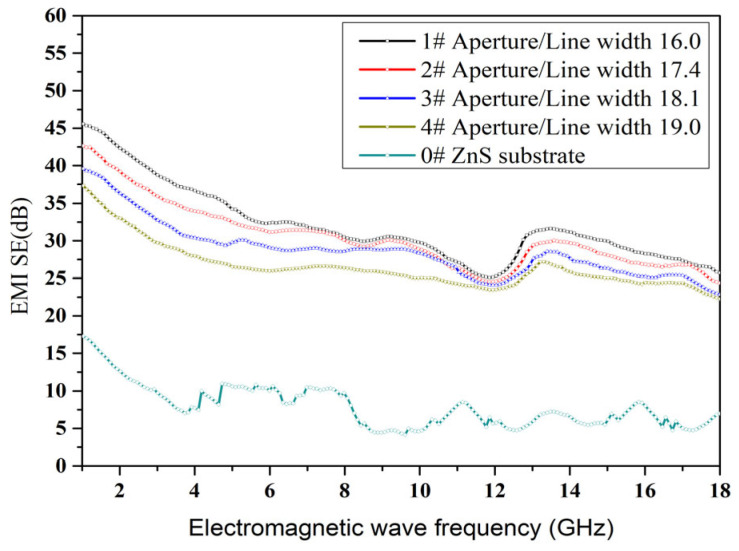
EMI SE of the ZnS optical windows with the Au mesh.

If the substrate of the optical window does not have the ability to dissipate electromagnetic waves, then achieving electromagnetic shielding function mainly depends on the reflection loss of the metal mesh on the substrate surface. According to Equation (2) in Section 2, the reflection loss is inversely proportional to the surface resistance. The smaller the surface resistance, the greater the electromagnetic shielding effectiveness. Conversely, the larger the surface resistance, the smaller the electromagnetic shielding effectiveness. The Au mesh with the 16.0 ratio of the mesh aperture and line width achieved the highest average EMI SE value of 33.2 dB in the 1–18 GHz band. This result is also consistent with the surface resistance test results. Among the four experimental samples, the sample with a ratio of 16.0 has the smallest surface resistance value. Within the frequency of 1–18 GHz, the EMI SE of all samples is higher than 22.5 dB (Figure 9). As the frequency increases from 1 to 18 GHz, the EMI SE should gradually decrease, However, there are fluctuations in electromagnetic shielding effectiveness at 14 GHz, which are caused by system testing errors. To illustrate the effectiveness and potential applications of depositing random structured metal mesh shielding films on ZnS substrates, a comparison of several metal metals [41,42,43,44,45] with similar geometries but different fabrication processes is presented in Table 1.

## 5. Conclusions

We have created four irregular structure metal-mesh films on the ZnS glass with excellent microwave shielding performance, based on different aperture and line width ratios. Our findings indicate that the Au mesh film retains the exceptional light transmission characteristics of random-pattern metal meshes, achieving high IR transmittance (~60%) and a broad transmission spectrum (2–12 μm). We analyzed the surface resistance of four types of metal mesh with different *g*/*w*, and the samples with higher coverage have lower surface resistance. For example, surface resistance was 16.7 Ω/sq for Au meshes with the coverage ratio 11.64%; however, a surface resistance of 9.5 Ω/sq corresponds to a coverage ratio of 15.33%. It exhibited superior EMI shielding performance in the wide frequency of 1–18 GHz (SE > 22.5 dB). The experimental results demonstrated that the theoretical calculations and designs of the structural parameters of metal mesh on ZnS substrates were scientific and effective.

## Figures and Tables

**Table 1:** Comparison of various electromagnetic shielding films on the ZnS subtrate.

Subtrate	Films Material and Morphology	Grid Structure	Transmittance	Sheet Resistances	EMI SE	Refs.
ZnS (thickness 4.5 mm)	Double layer films: Cu radom grid	line width: 1.75–1.96 μm, average period: 50–106 μm	460–800 nm and 8–12 μm: >89.8%	85 Ω/sq	2–18 GHz: >31 dB	[41]
ZnS	Ag period grid	period: 7.62 mm, line width: 0.635 mm	8–12 μm: >80%	—	1 GHz: 23.3	[42]
ZnS	WSi_2_ single layer	Thickness 0.75 μm	—	—	0.4–18 GHz: >30 dB	[43]
ZnS	Cu radom grid	period: 80–100 μm, line width: 2.3 μm thickness: 6.4 μm	550 nm: 71%, 3–10 μm: 65–72%	0.06–0.15 Ω/sq	—	[44]
ZnS	graphene layer, 13 layers	—	8–12 μm: 94.7%	68 Ω/sq	30 MHz~1.5 GHz: 15.6 dB	[45]
ZnS	single layer films: Au radom grid	The ratios of the aperture to line width were 16.1, 17.4, 18.0, and 19.0	2–10 μm: 57.1–63.2%	9.5–16.7 Ω/sq	1–18 GHz: >22.5 dB	This work

## Data Availability

The original contributions presented in this study are included in the article. Further inquiries can be directed to the corresponding authors.

## Supplementary Materials

The following supporting information can be downloaded at: https://www.mdpi.com/article/10.3390/ma18051067/s1.
