# Supplementary material for: Electromagnetic Interference (EMI) Shielding Performance and Photoelectric Characteristics of ZnS Infrared Window
Source: Materials (Basel). 2025 Feb 27;18(5):1067. doi: 10.3390/ma18051067 (PMC11901063; doi:10.3390/ma18051067)
Supplement: Supplementary file 1 [file materials-18-01067-s001.zip › materials-3407436-supplementary.pdf]

Article

Electromagnetic Interference (EMI) Shielding Performance and Photoelectric Characteristics of ZnS Infrared Window

Liqing Yang [2], Rongxing Guo [1], Fei Gao [2], Yongmao Guan [2], Mengwen Zhang [1,*] and Pengfei Wang [2,*]

[1] Henan key Laboratory of Aeronautical Materials and Application Technology, Zhengzhou University of Aeronautics, Zhengzhou 450046, China; guorongxing@zua.edu.cn

[2] National Key Laboratory of Ultrafast Optical Science and Technology, Xi'an Institute of Optics and Precision Mechanics, Chinese Academy of Sciences (CAS), Xi'an 710119, China; yangliqing@opt.ac.cn (L.Y.); gaofei1980@opt.ac.cn (F.G.); guanyongmao@opt.ac.cn (Y.G.)

* Correspondence: zhangmengwen@zua.edu.cn (M.Z.); pfwang@opt.ac.cn (P.W.); Tel.: +86-29-88887505 (P.W.)

The cracked template was prepared according to the following procedure. First, an initiator solution was obtained by dissolving 0.8 g of APS (80.0 g in ultrapure water). Meanwhile, 80.0 g Methyl methacrylate (MMA), 80.0 g Butyl acrylate (BA), 4.8 g 2-Hydroxypropyl acrylate (HPA), and 2.4 g AA (Acrylic acid) were combined in a beaker to obtain a monomer mixture. Then, an emulsified mixture was prepared by emulsifying 16.7 g of monomer mixture, 2.0 g of $NaHCO_3$, and an aqueous solution prepared by dissolving 4.8 g of OP-10 and 3.2 g of Sodium dodecyl sulfate (SDS) in 140 g of ultrapure water at 1000 rpm using a magnetic stirrer. Next, 16.2 g of initiator solution was added while blending continuously after the emulsified mixture had been heated to 75 °C. The mixture was heated to 80 °C and maintained for 30 min. Both the remaining monomer mixture and initiator solution were then added drop wise to the mixture at 80 °C with continuously stirring. The drop addition time for the initiator solution was 3 h, whereas that for the monomer mixture was slightly shorter than 3 h. Afterwards, the reaction mixture was kept at 85 °C for 2 h. After the reaction mixture cooled to room temperature, an appropriate amount of ammonia solution was added, and the pH was adjusted to 7.0–7.5. Finally, the reaction mixture was filtered three times through a filter cloth (400 × 400 mesh per inch) to obtain the crackle precursor.
